# Correction to: Disentangling environmental effects in microbial association networks

**DOI:** 10.1186/s40168-021-01209-4

**Published:** 2021-12-21

**Authors:** Ina Maria Deutschmann, Gipsi Lima-Mendez, Anders K. Krabberød, Jeroen Raes, Sergio M. Vallina, Karoline Faust, Ramiro Logares

**Affiliations:** 1 Institute of Marine Sciences, CSIC, Passeig Marítim de la Barceloneta, 37-49, 08003 Barcelona, Spain; 2 Research Unit in Biology of Microorganisms (URBM), University of Namur, 61 Rue de Bruxelles, 5000 Namur, Belgium; 3 Department of Biosciences/Section for Genetics and Evolutionary Biology (EVOGENE), University of Oslo, p.b. 1066, Blindern, N-0316 Oslo, Norway; 4 VIB Center for Microbiology, Herestraat 49-1028, 3000 Leuven, Belgium; 5 KU Leuven Department of Microbiology, Immunology and Transplantation, Rega Institute, Laboratory of Molecular Bacteriology, Herestraat 49, 3000 Leuven, Belgium; 6 Spanish Institute of Oceanography (IEO - CSIC), Ave Principe de Asturias 70 Bis, 33212 Gijon, Spain

**Correction to: Microbiome 9, 232 (2021)**

**https://doi.org/10.1186/s40168-021-01141-7**

Following the publication of the original article [1], the author reported that there is a discrepancy on the presentation of eq. 3 between the PDF and HTML. The PDF shows the correct presentation.

The original article has been updated to correct this.
