# Supplementary material for: Disentangling environmental effects in microbial association networks
Source: Microbiome. 2021 Nov 26;9:232. doi: 10.1186/s40168-021-01141-7 (PMC8620190; doi:10.1186/s40168-021-01141-7)
Supplement: Supplementary file 2 — Additional file 1: Table S1: Comparison between methods on correctly detecting false associations. We computed the fraction (in percentage) of correctly detected false associations for each of the 1000 simulated datasets. There are only few edges that are detected by only one approach (first four rows). The most prominent groupings are highlighted in grey, e.g., SP, OL, and II agree on average on a third of edges. Less prominent groupings are aggregated with others. Table S2: Performance of environmentally-driven edge detection methods on simulated networks These include 50 microorganisms and 1225 possible associations. Values display median (standard deviation) for simulated networks and simulated networks incorporating noise. Combi refers to intersection combination of all four methods. The methods with highest or lowest median, respectively, are indicated with an asterisk. Table S3 Number of triplets an microbial edge is part of in the BBMO network. SP and OL not listed below because they remove 100% of microbial associations that are within at least one triplet. Table S4 The BBMO network based on real data It contained bacteria and eukaryotes from the picoplankton and nanoplankton. This table summarizes the number and fractions of microbial associations classified by EnDED as environmentally-driven. Combi refers to the intersection combination of all four methods, II to Interaction Information, and DPI to Data Processing Inequality. Both methods, sign Pattern and Overlap, are not shown because both remove all microbial edges found in at least one triplet. For example, 349 (14.9%) associations between bacteria from the picoplankton with eukaryotes from the nanoplankton were classified by intersection combination as environmentally-driven (indirect), II classified 30.6% and DPI 37.2% as environmentally-driven. [file 40168_2021_1141_MOESM2_ESM.docx]

Additional Files

Additional File 1

**Supplementary Table S1: Comparison between methods on correctly detecting false associations**. We computed the fraction (in percentage) of correctly detected false associations for each of the 1000 simulated datasets. There are only few edges that are detected by only one approach (first four rows). The most prominent groupings are highlighted in grey, e.g., SP, OL, and II agree on average on a third of edges. Less prominent groupings are aggregated with others.

| **Statistic** | **Minimum** | **1^st^ Quartile** | **Median** | **Mean** | **2^nd^ Quartile** | **Maximum** |
| --- | --- | --- | --- | --- | --- | --- |
| SP | 0 | 0 | 0.2 | 0.3 | 0.5 | 3.7 |
| OL | 0 | 0 | 0.1 | 0.2 | 0.3 | 2.0 |
| II | 0 | 0.7 | 1.3 | 1.4 | 2.0 | 6.0 |
| DPI | 0 | 0.1 | 0.3 | 0.4 | 0.6 | 2.6 |
| SP and OL | 4.9 | 12.2 | 14.9 | 15.0 | 17.5 | 30.0 |
| SP, OL, and II | 19.1 | 29.5 | 32.6 | 32.8 | 36.2 | 49.6 |
| SP, OL, and DPI | 2.6 | 7.1 | 8.9 | 9.1 | 10.8 | 22.1 |
| SP, OL, II, DPI, and COMBI | 22.4 | 32.1 | 35.6 | 35.5 | 38.6 | 48.6 |
| other | 0.4 | 3.3 | 4.9 | 5.1 | 6.6 | 15.4 |

Additional File 2

**Table S2:** **Performance of environmentally-driven edge detection methods on simulated networks** These include 50 microorganisms and 1225 possible associations. Values display median (standard deviation) for simulated networks and simulated networks incorporating noise. Combi refers to intersection combination of all four methods. The methods with highest or lowest median, respectively, are indicated with an asterisk.

| **Method** | **Combi** | **SP** | **OL** | **II** | **DPI** |
| --- | --- | --- | --- | --- | --- |
| without noise  number of nodes | 50 (0.045) | 47 (6.6) | 48 (5.6) | 50 (0.94) | 50 (0.1) |
| number of edges | 737 (50) | 140 (52) | 144 (58) | 354 (67) | 601 (60) |
| TP | 332 (47) | 893 (64)* | 888 (69) | 696 (72) | 459 (53) |
| TN | 45 (5.1)* | 8 (4.3) | 9 (4.7) | 24 (5.8) | 37 (5.5) |
| FP | 15 (4.6)* | 51 (5.8) | 51 (6.2) | 36 (6.4) | 23 (5.2) |
| FN | 692 (48) | 131 (49)* | 136 (54) | 330 (63) | 564 (56) |
| TPR | 0.32 (0.04) | 0.87 (0.05)* | 0.87 (0.05)* | 0.68 (0.06) | 0.45 (0.05) |
| TNR | 0.75 (0.07)* | 0.14 (0.07) | 0.15 (0.08) | 0.4 (0.10) | 0.62 (0.08) |
| FPR | 0.25 (0.07)* | 0.86 (0.07) | 0.85 (0.08) | 0.6 (0.10) | 0.38 (0.08) |
| PPV | 0.96 (0.011)* | 0.95 (0.005) | 0.95 (0.005) | 0.95 (0.007) | 0.95 (0.009) |
| ACC | 0.35 (0.04) | 0.83 (0.04)* | 0.83 (0.048)* | 0.66 (0.057) | 0.46 (0.046) |
| with noise  number of nodes | 50 (0.08) | 47 (5.6) | 48 (4.9) | 50 (0.47) | 50 (0.12) |
| number of edges | 828 (56) | 144 (53) | 149 (59) | 428 (79) | 717 (73) |
| TP | 219 (48) | 864 (69)* | 860 (72) | 605 (81) | 324 (64) |
| TN | 49 (5)* | 9 (4.6) | 9 (4.9) | 29 (6.3) | 42 (5.8) |
| FP | 10 (3.9)* | 50 (6.1) | 50 (6.4) | 30 (6.6) | 17 (5.1) |
| FN | 779 (53) | 137 (50)* | 139 (55) | 398 (75) | 674 (69) |
| TPR | 0.22 (0.05) | 0.86 (0.05)* | 0.86 (0.06)* | 0.6 (0.08) | 0.32 (0.06) |
| TNR | 0.84 (0.07)* | 0.15 (0.08) | 0.16 (0.08) | 0.49 (0.1) | 0.72 (0.09) |
| FPR | 0.16 (0.07)* | 0.85 (0.08) | 0.84 (0.08) | 0.51 (0.1) | 0.28 (0.09) |
| PPV | 0.96 (0.014)* | 0.95 (0.005) | 0.95 (0.005) | 0.95 (0.007) | 0.95 (0.012) |
| ACC | 0.25 (0.04) | 0.82 (0.05)* | 0.82 (0.05)* | 0.6 (0.07) | 0.34 (0.06) |

SP - Sign Pattern; OL - Overlap; II - Interaction Information; DPI - Data Processing Inequality; Combi-intersection combination

Additional file 3

**Table S3** **Number of triplets an microbial edge is part of in the BBMO network.** SP and OL not listed below because they remove 100% of microbial associations that are within at least one triplet.

| **Triplets** | **all** | **pos (%)** | **neg (%)** | **Combi (%)** | **II (%)** | **DPI (%)** |
| --- | --- | --- | --- | --- | --- | --- |
| 0 | 4 590 | 4 124 (89.8) | 466 (10.2) | NA | NA | NA |
| 1 | 16 193 | 13 369 (82.6) | 2 824 (17.4) | 1 276 (7.9) | 3 851 (23.8) | 4 560 (28.2) |
| 2 | 8 266 | 6 404 (77.5) | 1 862 (22.5) | 1 048 (12.7) | 3 335 (40.3) | 2 585 (31.3) |
| 3 | 667 | 484 (72.6) | 183 (27.4) | 140 (21.0) | 388 (58.2) | 222 (33.3) |
| 4 | 81 | 56 (69.1) | 25 (30.9) | 22 (27.2) | 75 (92.6) | 25 (30.9) |
| 5 | 22 | 20 (90.9) | 2 (9.1) | 2 (9.1) | 22 (100) | 2 (9.1) |
| 6 | 1 | 1 (100) | NA | NA | 1 (100) | NA |

Additional file 4

**Table S4** **The BBMO network based on real data** It contained bacteria and eukaryotes from the picoplankton and nanoplankton. This table summarizes the number and fractions of microbial associations classified by EnDED as environmentally-driven. Combi refers to the intersection combination of all four methods, II to Interaction Information, and DPI to Data Processing Inequality. Both methods, Sign Pattern and Overlap, are not shown because both remove all microbial edges found in at least one triplet. For example, 349 (14.9%) associations between bacteria from the picoplankton with eukaryotes from the nanoplankton were classified by intersection combination as environmentally-driven (indirect), II classified 30.6% and DPI 37.2% as environmentally-driven.

| **Type** | **edges** | **positive** | **negative** | **triplets** | **Combi** | **II** | **DPI** |
| --- | --- | --- | --- | --- | --- | --- | --- |
| nB | 6 377 | 5 453 (85.5) | 924 (14.5) | 5 150 (80.8) | 376 (5.9) | 1 512 (23.7) | 1 080 (16.9) |
| n+pB | 5 191 | 4 069 (78.4) | 1 122 (21.6) | 4 824 (92.9) | 440 (8.5) | 1 381 (26.6) | 1 678 (32.3) |
| pB | 2 832 | 2 053 (72.5) | 779 (27.5) | 2 160 (76.3) | 125 (4.4) | 569 (20.1) | 631 (22.3) |
| nE | 1 319 | 1 163 (88.2) | 156 (11.8) | 1 016 (77.0) | 113 (8.6) | 350 (26.5) | 254 (19.3) |
| n+pE | 1 165 | 976 (83.8) | 189 (16.2) | 1 006 (86.4) | 158 (13.6) | 353 (30.3) | 370 (31.8) |
| pE | 895 | 820 (91.6) | 75 (8.4) | 543 (60.7) | 44 (4.9) | 153 (17.1) | 113 (12.6) |
| nB+E | 4 703 | 4 080 (86.8) | 623 (13.2) | 4 120 (87.6) | 438 (9.3) | 1 345 (28.6) | 1 043 (22.2) |
| pB+E | 2 520 | 1 908 (75.7) | 612 (24.3) | 1 980 (78.6) | 204 (8.1) | 626 (24.8) | 647 (25.7) |
| nB+pE | 2 483 | 2 100 (84.6) | 383 (15.4) | 2 222 (89.5) | 241 (9.7) | 668 (26.9) | 709 (28.6) |
| pB+nE | 2 335 | 1 836 (78.6) | 499 (21.4) | 2 209 (94.6) | 349 (14.9) | 715 (30.6) | 869 (37.2) |

B - Bacteria; E - Eukaryotes; n - nano fraction; p - pico fraction
